# White matter hyperintensities are associated with locus coeruleus atrophy and astrocytic β_2_‐adrenergic receptor expression

**DOI:** 10.1002/alz.71583

**Published:** 2026-06-17

**Authors:** Victor Vidal, Gonzalo Farías, Carolina Delgado, Paul H. Delano, Rodrigo C. Vergara, Patricia Orellana, Tomás Ossandón, Nicolás A. Crossley, Cecilia Gonzalez‐Campo, Sharon L. Naismith, Raul Gonzalez‐Gomez, Carlos Coronel‐Oliveros, Agustín Ibáñez, Gabriel Wainstein, Robert D. Sanders, James M. Shine, Vicente Medel

**Affiliations:** ^1^ Brain and Mind Centre The University of Sydney Sydney Australia; ^2^ Departamento de Neurociencia Facultad de Medicina Universidad de Chile Santiago Chile; ^3^ Hospital Clínico de la Universidad de Chile Santiago Chile; ^4^ Departamento de Kinesiología Universidad Metropolitana de Ciencias de la Educación Santiago Chile; ^5^ Centro Nacional de Inteligencia Artificial (CENIA) Santiago Chile; ^6^ Departamento de Psiquiatría Facultad de Medicina Pontificia Universidad Católica de Chile Santiago Chile; ^7^ Institute for Biological and Medical Engineering, Schools of Engineering, Medicine and Biological Sciences Pontificia Universidad Católica de Chile Santiago Chile; ^8^ Centro de Neurociencia Cognitiva Universidad de San Andrés Buenos Aires Argentina; ^9^ Latin American Brain Health Institute (BrainLat) Universidad Adolfo Ibanez Santiago Chile; ^10^ Global Brain Health Institute (GBHI) University of California, US Trinity College Dublin Dublin Ireland; ^11^ Department of Biophysics School of Medicine Istanbul Medipol University Istanbul Turkey; ^12^ Barcelonaβeta Brain Research Center (BBRC) Pasqual Maragall Foundation Barcelona Spain; ^13^ Facultad de Ciencias Biológicas Pontificia Universidad Católica de Chile Santiago Chile

**Keywords:** β_2_‐adrenergic receptors, aging, locus coeruleus, metabolism, white matter hyperintensities

## Abstract

**INTRODUCTION:**

White matter hyperintensities (WMHs) are a robust marker of brain aging and dementia risk, typically attributed to vascular pathology. However, impaired astrocytic support may also contribute. The locus coeruleus (LC), which degenerates early in aging and Alzheimer's disease, provides widespread noradrenergic projections that regulate astrocytic metabolism via β_2_‐adrenergic signaling.

**METHODS:**

In a healthy aging cohort (*N* = 106), we quantified LC volume, WMHs, and voxel‐wise (q‐WMHs). Associations were tested, controlling for age, cardiovascular risk, and other subcortical nuclei. Spatial partial least squares regression related q‐WMH patterns to LC volume, age, and cortical adrenergic receptor expression.

**RESULTS:**

LC volume was independently associated with WMH burden and mediated age‐related WMH increases. A latent q‐WMH pattern aligned with cortical β_2_‐adrenergic receptor expression and mediated its association with WMH burden.

**DISCUSSION:**

LC degeneration may contribute to regional WMH vulnerability through noradrenergic mechanisms consistent with astrocytic β_2_‐adrenergic signaling, highlighting a potential nonvascular pathway influencing white matter integrity.

## Highlights

- Locus coeruleus (LC) atrophy predicts white matter hyperintensity (WMH) burden in aging.
- LC atrophy partially mediates the association between age and WMH burden.
- WMH spatial patterns align with cortical β_2_‐adrenergic receptor (ADRB2) expression gradients.
- LC atrophy increases coupling between WMH distribution and ADRB2 expression maps.
- Findings support a spatial vulnerability gradient linked to noradrenergic regulation of white matter.

## 1 BACKGROUND

White matter hyperintensities (WMHs) are among the most robust neuroimaging markers of brain aging, consistently associated with cognitive decline, neuropsychiatric symptoms, and increased risk for neurodegenerative disease.^1^, ^2^, ^3^, ^4^ While traditionally interpreted as a consequence of vascular pathology, accumulating evidence suggests that WMHs can also reflect disruptions in brain metabolism,^2^, ^5^, ^6^, ^7^, ^8^ including impaired support for myelinated axons.^9^ However, the upstream mechanisms underlying this vulnerability remain poorly understood.

Recent work has shown that white matter regions susceptible to lesion formation exhibit distinct metabolic profiles, with reduced glycolysis in healthy individuals and compensatory glycolysis in those with small vessel disease.^9^, ^10^ These patterns suggest an imbalance between energy demand and metabolic supply.^11^ One candidate modulator of this balance is the locus coeruleus (LC), a brainstem nucleus that degenerates early in aging and Alzheimer's disease.^12^, ^13^, ^14^ LC atrophy is thought to reflect progressive degeneration of noradrenergic neurons and reduced capacity for cortical noradrenaline release. Whether this same relationship applies to healthy aging remains uncertain.^15^ The LC influences cortical function through multiple adrenergic pathways, modulating neural excitability via α_2_‐adrenergic receptors (ADRA2A)^16^, ^17^, ^18^ and astrocytic function via α_1_‐ (ADRA1A) and β_2_‐adrenergic receptors (ADRB2).^19^, ^20^, ^21^, ^22^ Evidence suggests that β2 activation can support axonal energy demands by promoting glycolytic activity and lactate export from astrocytes, a process disrupted in small vessel disease.^23^, ^24^, ^25^, ^26^, ^27^

We hypothesize that LC atrophy is associated with disruption of a neuromodulatory–metabolic axis that contributes to regional vulnerability of cortical white matter. Specifically, we predict that reduced LC integrity is linked to increased WMH burden and to a characteristic spatial pattern of WMHs that aligns with cortical ADRB2 expression. Within this framework, WMHs may reflect the interaction between neurodegenerative processes and impaired noradrenergic modulation of brain metabolism, rather than arising solely from vascular mechanisms.

To evaluate this hypothesis, we analyzed structural magnetic resonance imaging (MRI) from a healthy aging cohort of older Chilean adults, in conjunction with cortical gene expression data from the Allen Human Brain Atlas. We combined manual and automated WMH segmentation with volumetric estimates of the LC while controlling for confounds, including age, cardiovascular risk, and volumes of the whole brainstem and other neuromodulatory nuclei. We mapped the cortical distribution of WMHs and tested their spatial correspondence with adrenergic receptor expression, focusing on β_2_‐receptors. Finally, through latent variable analyses, we assessed whether individual differences in the topographic loading of WMH and LC volume were associated with the cortical ADRB2 gradient. Together, these analyses provide a systems‐level framework linking LC degeneration with regionally specific WMH vulnerability in aging, consistent with a role for astrocytic β_2_‐adrenergic signaling.

## 2 METHODS

**Participants**: Participants were drawn from the Auditory and Dementia Study (ANDES^28^), a multidisciplinary cohort of cognitively unimpaired older adults in Chile. Inclusion criteria were age ≥65 years, Mini‐Mental State Examination (MMSE) score ≥ 24. Exclusion criteria included severe hearing loss, neurological disease, functional dependence, history of stroke or traumatic brain injury, significant psychiatric illness, otologic pathology, or severe sensory deficits interfering with cognitive testing. The final sample comprised 106 participants (mean age = 73.7 ± 5.5 years), of whom 65 were female. All participants provided written informed consent following the Declaration of Helsinki, and the study was approved by the local Institutional Review Board.

**MRI acquisition and preprocessing**: Structural MRI was acquired using a 3T Siemens MAGNETOM Skyra scanner with a 32‐channel head coil. Sequences included a T1‐weighted 3D magnetization prepared rapid gradient echo  (MPRAGE; repetition time [TR] = 2300 ms, echo time [TE] = 2.32 ms, flip angle = 8°, voxel size = 0.94 × 0.94 × 0.9 mm^3^) and a T2‐weighted fluid‐attenuated inversion recovery (FLAIR; TR = 8000 ms, TE = 94 ms, inversion time [TI] = 2500 ms) acquired using a 2D inversion‐recovery turbo spin‐echo sequence (TR = 8000 ms, TE = 94 ms, TI = 2500 ms) with a slice thickness of 3 mm, slice spacing of 3.6 mm, and an acquisition matrix of approximately 320 × 224. T1 and FLAIR images were converted from DICOM to NIfTI format using MRIcroGL. T1‐weighted images were processed using FreeSurfer v6.0 for brain segmentation and cortical parcellation, yielding estimates of total intracranial volume (eTIV), brainstem volume, gray and white matter volumes, and regional parcellations based on the Desikan–Killiany atlas. All images were manually inspected to ensure alignment with the anterior commissure. Voxel‐based morphometry (VBM) was conducted using SPM12 and the DARTEL pipeline to estimate gray matter density.

**LC and subcortical volume estimation**: LC volume was estimated using the MetaMask, a validated probabilistic atlas derived from neuromelanin‐sensitive MRI templates aligned to postmortem histology.^29^ This atlas provides an anatomical prior for LC localization and has been used to approximate LC structure using conventional T1‐weighted MRI in VBM analyses. The MetaMask was registered to the Montreal Neurological Institute (MNI) space and thresholded to balance anatomical specificity and intersubject robustness. The resulting LC mask contained 76 voxels (41 left, 35 right) at the resolution of the normalized images. The mask was applied to each participant's normalized, modulated gray matter maps derived from VBM preprocessing in SPM12 using the DARTEL pipeline (1‐mm isotropic resolution). After template generation, spatial normalization, and modulation, gray matter maps were smoothed using a 2‐mm full‐width at half‐maximum (FWHM) Gaussian kernel.^30^

**RESEARCH IN CONTEXT**

1. **Systematic review**: We reviewed the literature using PubMed and related databases, focusing on white matter hyperintensities (WMHs), neuromodulatory subcortical systems, and Alzheimer's disease. Previous studies have primarily attributed WMHs to vascular mechanisms, while independent lines of research have demonstrated early degeneration of the locus coeruleus (LC) and its role in regulating astrocytic and metabolic support processes. However, these frameworks have rarely been integrated into human studies of WMHs.
2. **Interpretation**: Our findings provide in vivo human evidence that LC atrophy is associated with both the burden and spatial distribution of WMHs. Importantly, WMH spatial patterns align with cortical β_2_‐adrenergic receptor expression gradients, suggesting a spatially structured vulnerability linked to noradrenergic regulation. These results extend current models of WMH pathophysiology beyond purely vascular explanations, pointing to a contribution of neuromodulatory systems to regional white matter vulnerability.
3. **Future directions**: Future research should investigate whether LC‐related neuromodulatory processes contribute to WMH progression and cognitive decline using longitudinal and multimodal approaches. Studies incorporating neuromelanin‐sensitive magnetic resonance imaging (MRI), molecular imaging, and subject‐specific measures of neuromodulatory function will be critical to clarify the mechanisms underlying these associations and their potential clinical relevance.

To account for individual differences in brain size, LC estimates were adjusted for eTIV. Total brainstem volume was included as a separate covariate in regression models to control for regional brainstem atrophy. Volumes of additional subcortical neuromodulatory nuclei were estimated using the same VBM‐based approach, including the substantia nigra^31^ (SN) and the basal forebrain cholinergic system.^32^ All volumes were extracted from the same modulated gray matter maps and adjusted for eTIV. As an additional anatomical specificity control, we defined a dorsal pontine control region of interest (ROI) adjacent to the LC^29^ and extracted tissue signals using the same VBM‐based procedure. To further evaluate the robustness of the LC definition, sensitivity analyses were conducted using eroded and dilated versions of the LC mask generated through morphological operations (see Supplementary Methods).

**WMH assessment**: WMHs were assessed using manual clinical rating scales and automated segmentation methods to ensure robustness and cross‐validation. First, manual ratings were performed on T2‐weighted FLAIR images by two independent trained experts (C.D., P.O.) following established visual scales. Global and regional burden of WMHs was rated using the age‐related white matter changes (ARWMC^33^) scale and the Fazekas scale,^34^ which separately score periventricular and deep WMHs. Second, automated WMH segmentation was conducted using the Lesion Segmentation Toolbox (LST,^35^ version 2.0.15) implemented in SPM12. The Lesion Growth Algorithm (LGA) was applied to co‐registered T1‐weighted and FLAIR images, with an initial κ‐threshold of 0.3 to define seed voxels for lesion expansion. This approach produces individual probabilistic lesion maps, from which total WMH volumes (in mm^3^) and voxel‐wise lesion probabilities were extracted.

Manual and automated approaches were integrated by using visual rating scales as an external clinical benchmark to validate the automated q‐WMH estimates. Automated segmentation outputs were then used for all subsequent quantitative and spatial analyses. To assess convergence between methods, total WMH volumes derived from LST were compared against ARWMC and Fazekas scores using Spearman correlation. Given the strong correspondence between approaches, LST‐derived WMH measures were selected as the primary outcome for regression and spatial analyses, due to their continuous nature and suitability for voxel‐wise modeling. Automated segmentations were also visually inspected for quality control, and any misclassified scans were flagged and reprocessed when necessary.

**Cortical white matter parcellation and spatial q‐WMH mapping**: To localize WMHs across cortical regions, we performed white matter parcellation in each subject's native anatomical space. Regional white matter segmentation was obtained from FreeSurfer's wmparc volume, which parcellates the white matter underlying 68 cortical regions (34 per hemisphere) according to the Desikan–Killiany^36^ atlas. We computed the lesion burden within each white matter parcel for each subject by intersecting the LST‐derived WMH probability map with the corresponding regional labels in wmparc. Regional WMH burden was calculated as the sum of lesion probabilities within each parcel, resulting in a 68‐dimensional spatial WMH vector per subject. These vectors were assembled into a subject‐by‐region matrix and submitted to principal component analysis (PCA) to identify shared spatial patterns of WMH distribution across individuals. The first principal component (PC1), capturing the dominant mode of variance in regional WMH burden, was retained for subsequent analyses.

**Gene expression analysis**: Cortical gene expression profiles were obtained from the Allen Human Brain Atlas to assess the spatial correspondence between WMH topography and noradrenergic receptor distribution. Expression data from six postmortem human brains were preprocessed and mapped to the 68 cortical regions of the Desikan–Killiany atlas using the *abagen* ^37^ toolbox. For each gene, microarray probe expression levels were first *z*‐score normalized across regions within each donor and then averaged across donors. In addition, inter‐regional expression values were intensity‐normalized using T1w/T2w cortical ratios to account for laminar biases and spatial variability in tissue composition, following the procedure described by Medel et al.^38^ We focused on three genes encoding adrenergic receptor subtypes relevant to LC signaling: ADRB2, ADRA1A, and ADRA2A. The resulting expression maps were then spatially correlated with the PC1 of subject‐level WMH distributions using Spearman's rank correlation. To account for spatial autocorrelation inherent to cortical maps, statistical significance was assessed using spin permutation testing (*n* = 1000 surface‐based rotations), implemented via the *neuromaps* ^39^ toolbox following the procedure of Alexander‐Bloch et al.^40^ Spin tests preserve the geodesic structure of cortical maps and provide null distributions for spatial correlations between parcellated brain phenotypes.

**Alzheimer's Disease Neuroimaging Initiative replication sample**: Participants from an independent replication sample from the Alzheimer's Disease Neuroimaging Initiative (ADNI) were selected from ADNI‐1, 2, 3 (*n* = 126) to approximate the age range and diagnostic composition of the primary cohort. Structural MRI data included T1‐weighted and FLAIR sequences acquired according to standardized ADNI MRI protocols. T1‐weighted images were obtained using 3D MPRAGE sequences with approximately 1‐mm isotropic resolution. FLAIR images were acquired to detect WMHs; ADNI‐2 used a 2D FLAIR sequence, whereas ADNI‐3 implemented a higher‐resolution 3D FLAIR acquisition designed to improve sensitivity to white matter lesions. All images were processed using the same pipeline applied to the ANDES cohort to estimate LC volume and quantify q‐WMH burden. ADNI participants provided written informed consent, and study procedures were approved by the institutional review boards of participating sites.

**Statistical analyses**: Statistical analyses were conducted in Python and MATLAB. Associations between LC volume and total WMH burden were assessed using partial Spearman correlations and multiple linear regression models. All covariates (age, sex, education, eTIV, Framingham Risk Score (FRS), and volumes of other neuromodulatory nuclei) were entered simultaneously into the main regression models to provide a conservative estimate of LC‐specific effects. To assess robustness given the sample size, key effects were additionally verified using reduced models and partial correlations, yielding consistent results. All continuous variables were *z*‐score standardized before analysis.

Mediation analyses were performed to test whether LC volume mediated the relationship between age and WMH burden. Indirect effects were estimated using 1000 bias‐corrected bootstrap samples, and confidence intervals were computed using the percentile method.

To capture multivariate topographic relationships between WMHs and neuroanatomical predictors, we applied partial least squares (PLS) regression. Voxel‐wise WMH probability maps (q‐WMHs) were vectorized for each subject and concatenated into a subject × voxel matrix (*X*), which was regressed onto a predictor matrix (*Y*) including LC, SN, basal forebrain cholinergic system, total brainstem volume, age, sex, education, eTIV, and FRS. All predictors were *z*‐scored to ensure comparability. PLS was implemented via singular value decomposition of the cross‐covariance matrix between *X* and *Y*. The first latent variable (PLS1), representing the dominant spatial mode of covariance, was retained for interpretation. Statistical significance of PLS components and predictor saliences was assessed using nonparametric permutation testing (10,000 iterations), which controls the family‐wise error rate at the component level.

For spatial correlation analyses involving cortical maps (PLS‐derived WMH patterns and adrenergic receptor expression), statistical significance was assessed using spin permutation tests (*n* = 1000 surface rotations). Where multiple spatial correlations were tested (e.g., across adrenergic receptor subtypes), *p*‐values were additionally corrected for multiple comparisons using false discovery rate (FDR) correction.

## 3 RESULTS

Table 1 summarizes the demographic and clinical characteristics of the study sample (*n* = 106). Participants were older adults (mean age 73 ± 5.46 years), with a higher proportion of women (64.2%) than men (35.8%), and an average of 11 ± 4.28 years of education. Cardiovascular risk, assessed using the FRS, was low to moderate on average (3.17 ± 1.85). WMH burden spanned a broad range, with mean Fazekas and ARWMC scores of 1.00 ± 0.84 and 4.86 ± 3.43, respectively.

**TABLE 1:** Demographic and neuropsychological assessment.

**Parameter**	**ANDES cohort (*n* = 106)**
Age	73 ± 5.46
Gender (M:F)	35.8:64.2
Education	11 ± 4.28
FRS	3.17 ± 1.85
WMHs Fazekas	1.00 ± 0.84
WMHs ARWMC	4.86 ± 3.43

Abbreviations: ANDES, Auditory and Dementia Study; ARWMC, age‐related white matter changes; F, female; FRS, Framingham Risk Score; M, male; WMH, white matter hyperintensity;

### 3.1 LC atrophy is associated with WMHs in older adults

**FIGURE 1:**
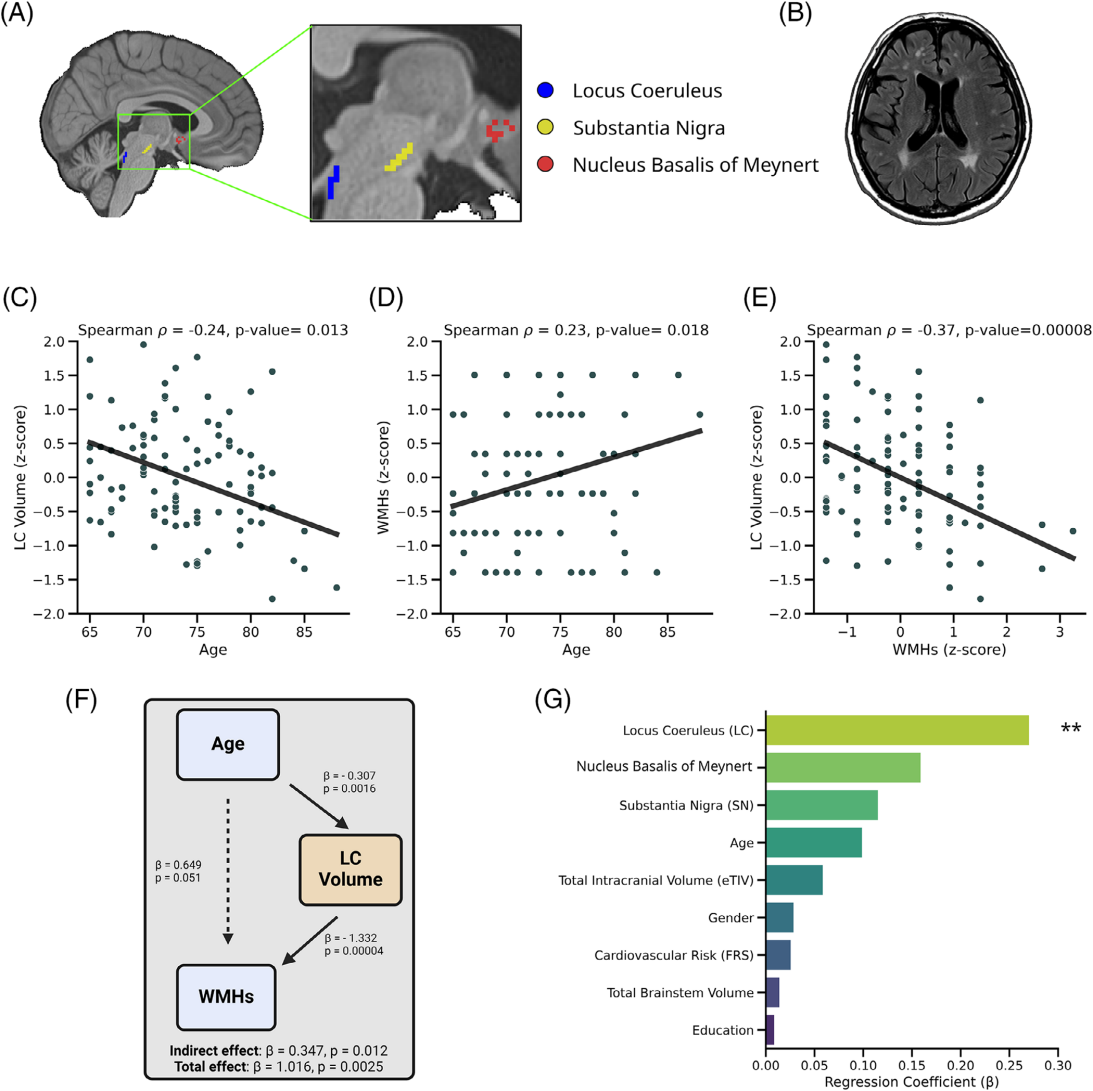
LC atrophy predicts WMH burden in aging. (A) Subcortical volume estimation via voxel‐based morphometry using MNI‐aligned probabilistic masks, including the LC. (B) Manual identification of WMHs on T2‐FLAIR MRI using the ARWMC scale. (C–E) Scatter plots showing partial Spearman correlations (controlling for eTIV between (C) age and LC volume (Spearman's *ρ* = −0.24, *p* = 0.013), (D) age and WMH burden (Spearman's *ρ* = 0.23, *p* = 0.018), and (E) LC volume and WMH burden (Spearman's *ρ *= −0.37, *p* = 0.00008). (F) Mediation model testing whether LC volume mediates the relationship between age and WMHs. The indirect path via LC was significant (*β* = 0.347, *p* = 0.012), while the direct effect of age became non‐significant when accounting for LC volume (*β* = 0.649, *p* = 0.051). (G) Multiple linear regression including LC, substantia nigra, basal forebrain, total brainstem volume, age, sex, education, and cardiovascular risk (Framingham Risk Score) as predictors of WMH burden. LC volume remained the only significant predictor (*β* = −0.27, *p* = 0.019; model *R* ^2^ = 0.20, *p* = 0.008). ARWMC, age‐related white matter changes; eTIV, estimated total intracranial volume; FLAIR, fluid‐attenuated inversion recovery; LC, locus coeruleus; MNI, Montreal Neurological Institute; MRI, magnetic resonance imaging; WMH, white matter hyperintensity.

We first examined bivariate associations among age, LC volume, and total WMH burden (Table 2). Age was negatively correlated with LC volume (Figure 1C, Spearman's *ρ* = −0.32, *p* = 0.0008) and positively associated with WMH burden (Figure 1D, Spearman's *ρ* = 0.20, *p* = 0.03). A partial correlation analysis further revealed a significant negative association between LC volume and total WMH burden, independent of total intracranial volume (Figure 1E, Spearman's *ρ* = −0.34, *p* = 0.00029).

**TABLE 2:** Multiple regression for WMHs (ARWMC).

**Parameter**	***β* (coef)**	**SE**	***p*‐value**
LC	−0.2701	0.118	0.024
SN	−0.1150	0.123	0.350
BF	−0.1588	0.116	0.176
Brainstem	−0.0138	0.045	0.759
eTIV	0.0584	0.114	0.610
Age	0.0986	0.108	0.365
Education	0.0085	0.023	0.712
Gender	−0.0284	0.260	0.913
FRS	−0.0255	0.102	0.803

Abbreviations: ARWMC, age‐related white matter changes; BF, basal forebrain; eTIV, estimated total intracranial volume; LC, locus coeruleus; SE, standard error; SN, substantia nigra; WMH, white matter hyperintensity.

To test the anatomical specificity of this relationship, we conducted a multiple linear regression model including volumes of other subcortical neuromodulatory structures with comparable scale and interindividual variability—namely the SN and the nucleus basalis of Meynert (NBM) cholinergic system (Ch4)—as well as total brainstem volume, age, sex, education, and cardiovascular risk (FRS). Although these regions share neuromodulatory roles and exhibit age‐related vulnerability, only LC volume uniquely explained variance in WMH burden in the full model (Table 2), and these effects were consistent when controlling for tissue segmentation of the subcortical ROIs (Table S1). Notably, LC volume accounted for variance in WMH burden beyond that shared with age or other subcortical nuclei, supporting a regionally specific association rather than a global subcortical or aging‐related effect (Figure 1G).

We evaluated the tissue composition of the LC mask to assess potential partial‐volume effects. The LC mask showed a mixed tissue profile across subjects, with white matter representing the largest fraction (51.8%), followed by gray matter (27.0%) and CSF (21.2%) (Figure S1A). LC gray matter fraction was modestly associated with age (Spearman's *ρ* = −0.24, *p* = 0.023), whereas LC white matter and CSF fractions were not (Figure S1C). LC gray matter estimates were not significantly correlated with local white matter or CSF estimates within the mask (Figure S1B). To test robustness to ROI definition, analyses were repeated using eroded and dilated LC masks (Supplementary Methods), yielding comparable effect sizes and direction of association with WMHs (eroded LC: *β* = −0.20, *p* = 0.054; dilated LC: *β* = −0.22, *p* = 0.035) (Figure S2). Finally, analyses using a dorsal pontine control ROI adjacent to the LC showed no significant associations between pontine tissue signals and WMH burden nor LC gray matter (Figure S3).

We next tested whether LC atrophy mediates the age‐related increase in WMH burden (Figure 1F). Mediation analysis revealed a significant indirect effect of age on WMHs through LC volume (*β *= 0.347, *p* = 0.012). In contrast, the direct impact of age on WMHs was attenuated and no longer statistically significant when controlling for LC volume (*β* = 0.649, *p* = 0.051). The total effect of age on WMHs remained significant (*β* = 1.016, *p* = 0.0025), indicating that individual differences in LC volume statistically account for a substantial portion of this association. These findings are consistent with a statistical mediation of the age‐–WMH relationship through LC structural variation.

### 3.2 Automated q‐WMH quantification

To assess the generalizability of the LC–WMH association, we performed an independent replication analysis using data from cognitively normal participants in the ADNI (*n* = 126). Using the same LC atlas and VBM‐based extraction procedure, LC gray matter estimates were derived from T1‐weighted images and tested against q‐WMH burden derived from FLAIR imaging. Consistent with the ANDES cohort, LC gray matter volume showed a significant negative association with q‐WMH burden in the ADNI cohort (Spearman's *ρ* = −0.26, *p* = 0.002; Figure S4B). In a multivariable regression model including age, sex, education, and intracranial volume, LC volume remained the strongest predictor of WMH burden (*β* = −0.34, *p* < 0.001; Figure S4A). These results replicate the LC–WMH association in an independent cohort with q‐WMHs.

**FIGURE 2:**
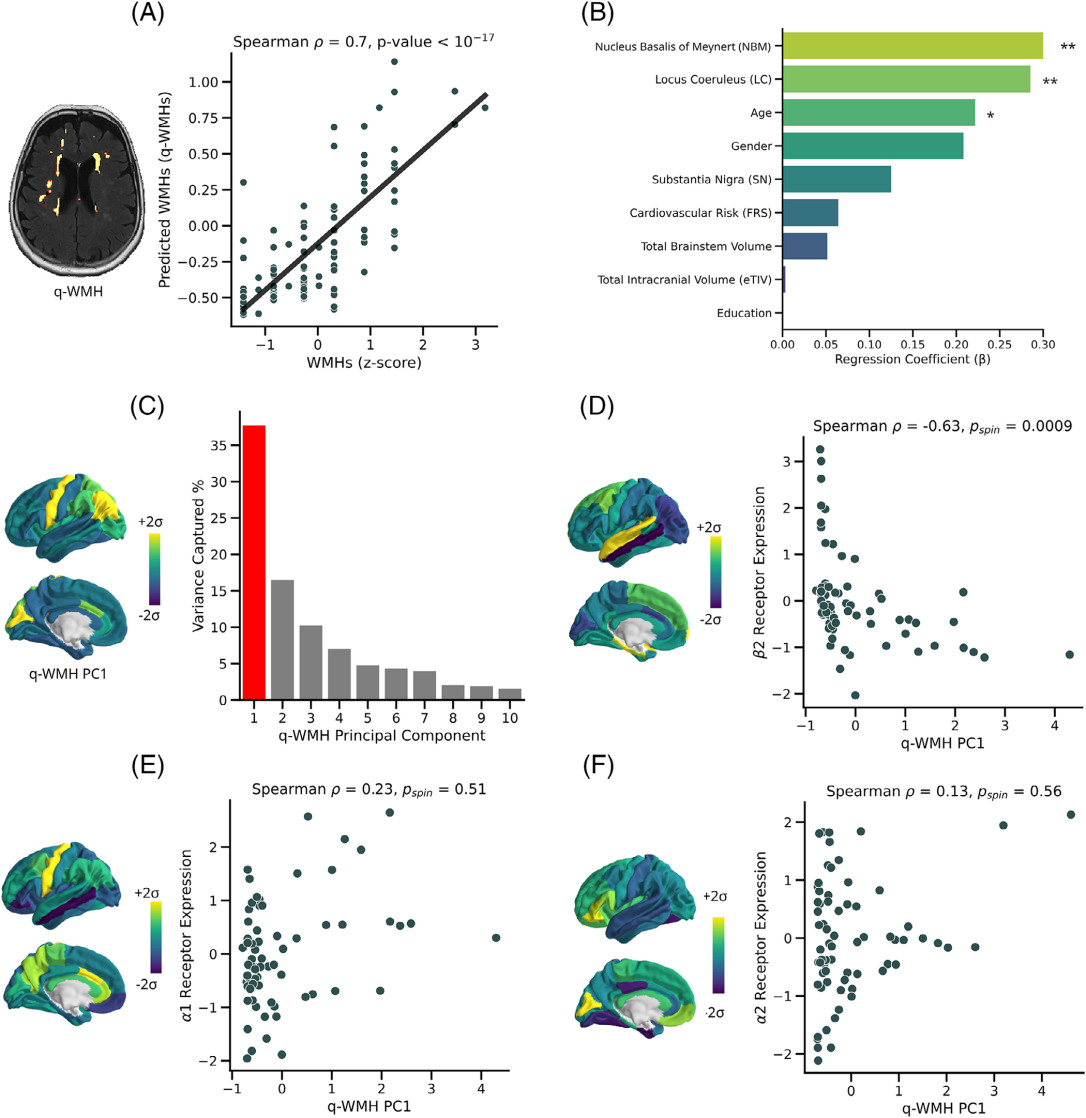
Spatial alignment of WMHs with β2‐adrenergic receptor expression. (A) Validation of q‐WMH using the LST. Automated q‐WMH volumes were strongly correlated with manual WMH ratings using the ARWMC scale (Spearman's *ρ* = 0.72, *p‐*value < 10^−^ ^17^). (B) Multiple linear regression predicting q‐WMH burden from anatomical and demographic variables. After controlling for subcortical volumes (substantia nigra, basal forebrain, brainstem), age, sex, education, total intracranial volume, and Framingham risk score, only LC volume, basal forebrain volume, and age remained significant predictors (LC *β* = −0.28, *p* = 0.006; NBM *β* = −0.3, *p *= 0.004; age *β* = 0.2, *p* = 0.02; model *R* ^2^ = 0.34, *p* < 10^−^^8^). (C) PCA of q‐WMH spatial maps across individuals. The PC1 explained 38% of the variance and represents the dominant spatial mode of WMH distribution. (D) Spatial correlation between q‐WMH PC1 and cortical ADRB2 expression derived from the Allen Human Brain Atlas (Spearman's *ρ *= −0.63, spin‐test *p* = 0.0009). No significant associations were observed for α_1_‐ or α_2_‐adrenergic receptor expression. ADRB2, β_2_‐adrenergic receptor; ARWMC, age‐related white matter changes; LC, locus coeruleus; LST, Lesion Segmentation Toolbox; NBM, nucleus basalis of Meynert; PC1, first principal component; PCA, principal component analysis; q‐WMH, automated white matter hyperintensity quantification; WMH, white matter hyperintensity.

To enable spatially resolved analyses, we used automated WMH probability maps derived from the LST. As a validation step, total WMH volumes obtained from automated segmentation showed significant correspondence with established visual rating scales, including the ARWMC scale (Spearman's *ρ *= 0.72, *p* < 10^−^^1^ ^7^) and the Fazekas scale (Spearman's *ρ* = 0.50, *p* = 3.7 × 10^−^^8^) (Figure 2A). While variability between methods was expected due to differences in measurement scale and resolution, this convergence supports the use of automated WMH estimates for quantitative and spatial analyses not achievable with ordinal visual ratings. The resulting subject‐specific q‐WMH maps were used for subsequent spatial and multivariate analyses. To test whether the relationship between LC atrophy and WMH burden generalized to this automated metric, we repeated the multiple linear regression model including volumes of the SN, NBM, total brainstem, eTIV, age, sex, education, and FRS as covariates (Figure 2B). In this model, LC volume, NBM volume, and age remained significantly associated with q‐WMH burden (LC β = −0.28, *p* = 0.006; NBM *β* = −0.3, *p* = 0.004; age *β* = 0.2, *p* = 0.02; model *R* ^2^ = 0.34, *p* < 10^−^^8^; Table 3), replicating the specificity of the LC–WMH link.

**TABLE 3:** Multiple regression for q‐WMHs (LST‐LGA).

**Parameter**	***β* (coef)**	**SE**	***p*‐Value**
LC	−0.2853	0.102	0.006
SN	−0.1250	0.106	0.241
BF	−0.3010	0.101	0.004
Brainstem	0.0515	0.039	0.189
eTIV	−0.0033	0.098	0.973
Age	0.2216	0.094	0.020
Education	0.0009	0.020	0.963
Gender	0.2082	0.225	0.356
FRS	0.0641	0.088	0.469

Abbreviations: BF, basal forebrain; eTIV, estimated total intracranial volume; FRS, Framingham Risk Score; LC, locus coeruleus; LGA, Lesion Growth Algorithm; LST, Lesion Segmentation Toolbox; q‐WMH, automated white matter hyperintensity quantification; SN, substantia nigra.

### 3.3 Spatial pattern of WMHs aligns with ADRB2 gene expression

To examine whether WMHs distribution follows patterns of adrenergic receptor gene expression, we assessed the spatial correspondence between q‐WMH burden and adrenergic receptor gene expression profiles. Using FreeSurfer's white matter parcellation (*wmparc*) based on the Desikan–Killiany atlas, we extracted regional q‐WMH loads across 68 cortical white matter parcels. A PCA across subjects revealed a dominant spatial mode (PC1), which accounted for 38% of the variance in WMH distribution (Figure 2C).

PC1 loading was significantly and negatively correlated with the cortical expression pattern of the ADRB2, as defined by the Allen Human Brain Atlas (Spearman's *ρ *= −0.63, spin‐test *p* = 0.0009; Figure 2D). No significant spatial associations were observed for ADRA1A (Figure 2E; Spearman's *ρ* = 0.2, spin‐test *p* = 0.51) or ADRA2A (Figure 2F; Spearman's *ρ* = 0.07, *p* = 0.72).

### 3.4 Multivariate associations between LC volume and the spatial distribution of q‐WMHs

**FIGURE 3:**
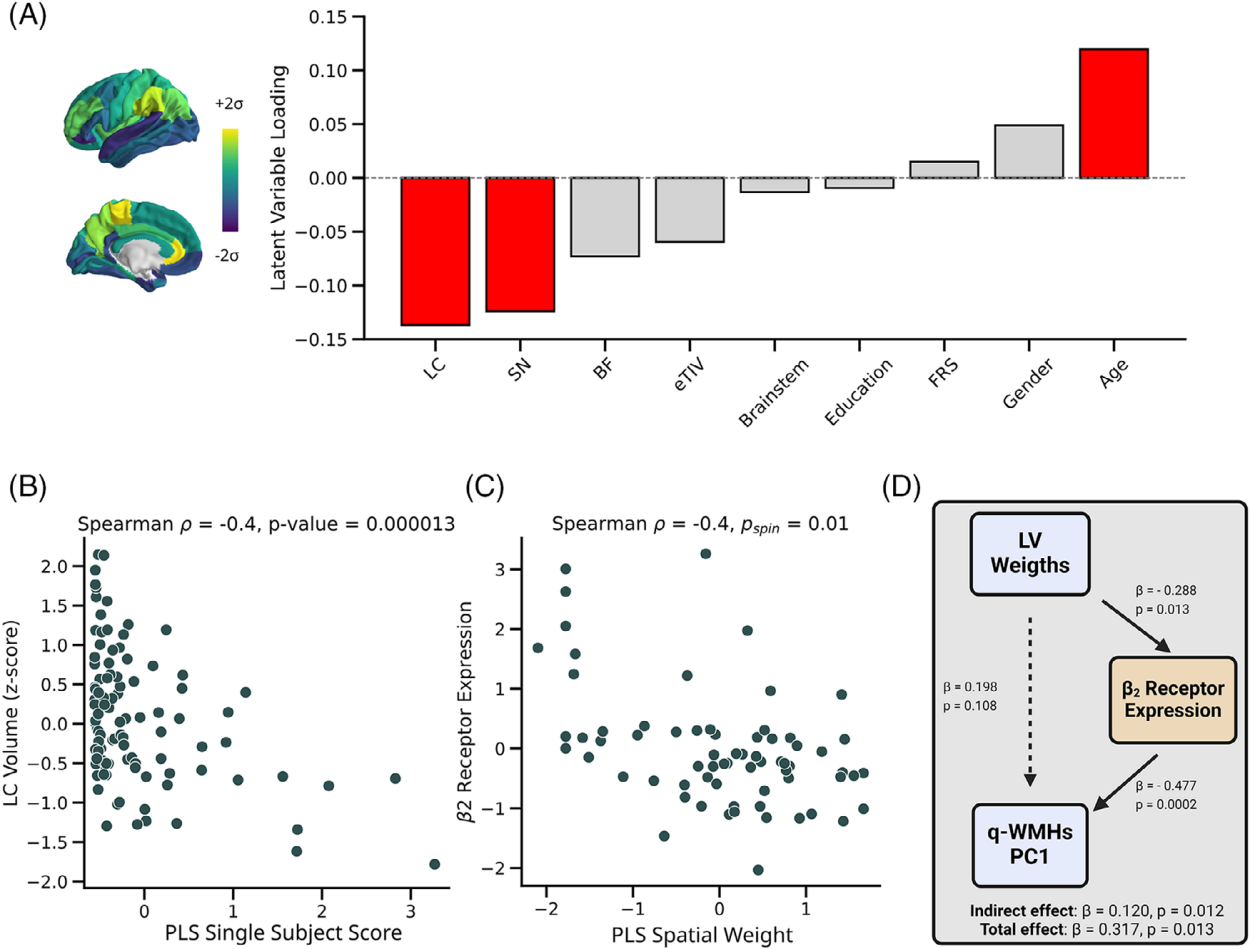
Multivariate spatial analysis of q‐WMHs burden reveals a key role for LC volume and ADRB2 expression. (A) PLS regression was used to model the parcel‐wise distribution of q‐WMHs as a function of individual anatomical and demographic predictors. The brain map shows the parcel‐wise spatial weights of the PLS1, which captures the dominant mode of covariance between predictors and WMH topography. Bar plots show PLS1 predictor loadings, with significant contributors (*p* < 0.05, permutation test 1000 iterations) highlighted in red. (B) At the individual level, LC volume was negatively associated with PLS1 single‐subject scores (Spearman's *ρ* = −0.40, *p* = 1.3 × 10^−^^5^), indicating that reduced LC volume is linked to greater expression of the PLS‐derived spatial pattern of WMHs. (C) The PLS1 spatial weight was significantly and negatively correlated with the regional expression of ADRB2s derived from the Allen Human Brain Atlas (Spearman's *ρ* = −0.4, spin‐test *p* = 0.01). (D) Spatial mediation analysis showed that the association between the PLS1 spatial pattern and the PC1 of q‐WMH maps was significantly mediated by ADRB2 expression, supporting a mechanistic link between noradrenergic signaling and regional white matter vulnerability. ADRB2, β2‐adrenergic receptor; LC, locus coeruleus; PC1, first principal component; PLS, partial least squares; PLS1, first latent variable; q‐WMH, automated white matter hyperintensity quantification.

To examine the relation between anatomical and demographic factors and the spatial distribution of q‐WMHs, we employed a multivariate PLS regression analysis. This approach identified a dominant spatial pattern that captured shared variation between individual q‐WMH maps and a set of anatomical and demographic predictors. The first latent component (PLS1) explained 24.7% of the variance in the spatial distribution of q‐WMHs. This component was significantly associated with LC volume, SN volume, and age (all *p* < 0.05; Figures 3A and S5). Among all predictors, LC volume showed the strongest contribution to this spatial pattern. These results indicate that LC atrophy is a major anatomical factor shaping the accumulation of WMHs across the cortex, exerting a stronger spatial influence than other neuromodulatory nuclei or demographic variables included in the model.

Subject‐level PLS1 scores, representing the degree to which each individual's q‐WMH map aligns with the latent spatial pattern, were negatively associated with LC volume (Spearman's *ρ* = −0.40, *p* = 1.3 × 10^−^^5^; Figure 3B). This indicates that individuals with lower LC volume exhibit stronger expression of the PLS‐derived q‐WMH spatial mode.

**FIGURE 4:**
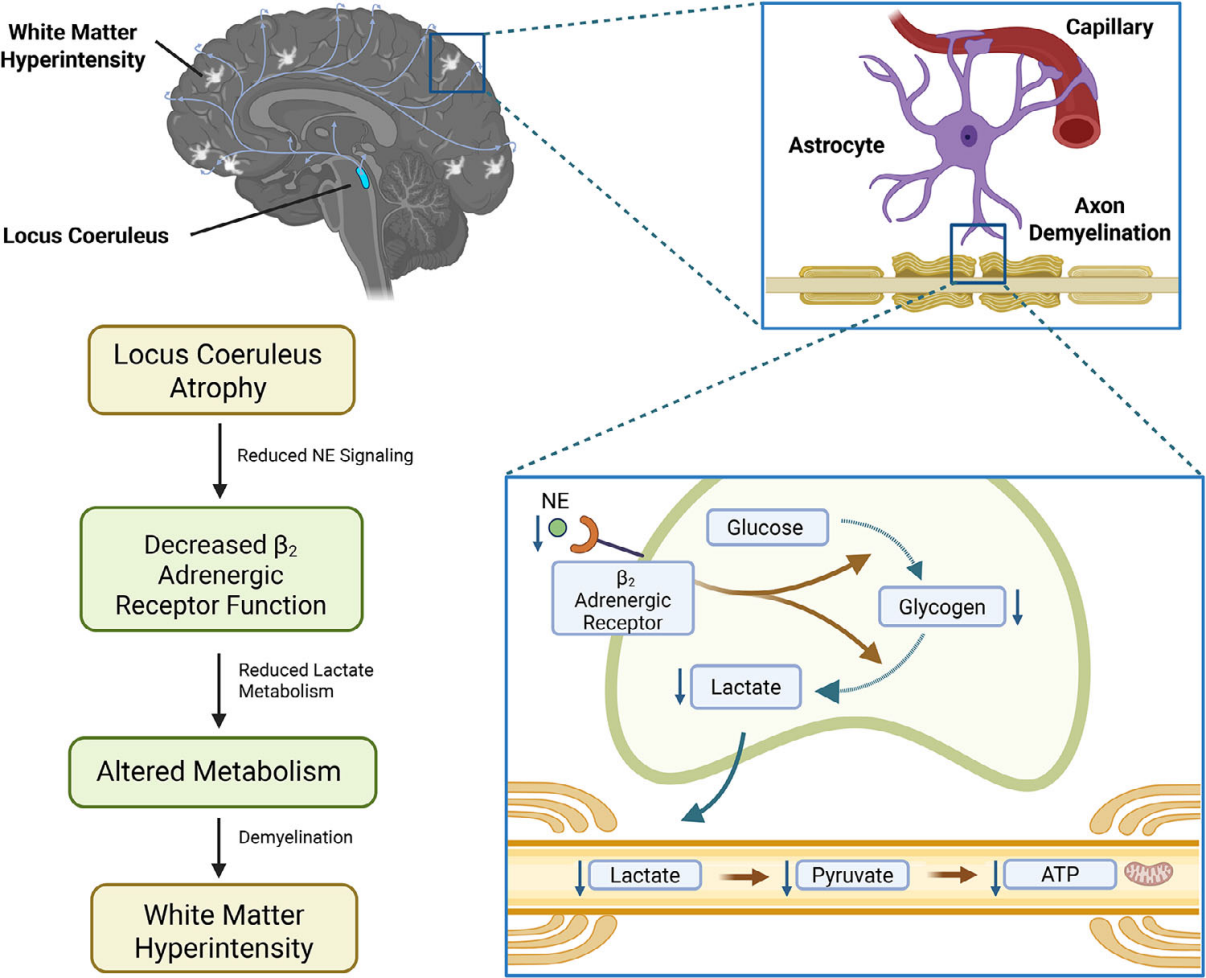
Mechanistic model linking LC degeneration to cortical white matter hyperintensities via astrocytic β_2_‐adrenergic signaling and metabolic support failure. LC atrophy reduces central noradrenergic tone, leading to diminished activation of astrocytic ADRB2s in distal cortical regions. This reduction impairs glycogenolysis and disrupts astrocyte‐neuron lactate shuttling, compromising the energy supply to myelinated axons. Over time, sustained metabolic insufficiency results in axonal demyelination and the emergence of focal WMHs. The schematic illustrates this spatially distributed cascade, emphasizing the interaction between neuromodulatory decline, astrocytic dysfunction, and white matter injury in aging. The model integrates our empirical findings of LC–WMH associations and their alignment with cortical β_2_‐receptor expression. ADRB2, β_2_‐adrenergic receptor; LC, locus coeruleus; WMH, white matter hyperintensity.

To assess the biological relevance of this spatial pattern, we correlated the parcel‐wise weights of PLS1 with the regional expression of ADRB2 expression from the Allen Human Brain Atlas. This revealed a significant spatial correspondence (Spearman's *ρ *= −0.40, spin‐test *p* = 0.011; Figure 3C), suggesting that regions with higher β_2_‐receptor expression are more vulnerable to WMHs in the context of LC atrophy. Finally, we tested whether the spatial similarity between the PLS‐derived pattern (PLS1) and the PCA‐derived q‐WMH pattern (PC1) was statistically mediated by β_2_‐receptor expression. A spatial mediation model (total effect *β* = 0.31, *p* = 0.013) revealed a significant indirect path via ADRB2 expression (*β* = 0.12, *p* = 0.012), while the direct effect became nonsignificant (*β* = 0.19, *p* = 0.108), consistent with a predominant indirect pathway (Figure 3D). Together, these results support a model in which LC structural integrity is linked to the spatial distribution of white matter damage, with regional vulnerability aligning with astrocytic ADRB2 expression (Figure 4).

## 4 DISCUSSION

Our findings support a model in which LC degeneration is closely linked to the accumulation of cortical WMHs in aging (Figure 4). LC atrophy was significantly associated with WMH burden, independent of age, cardiovascular risk, and other subcortical neuromodulatory structures. Importantly, this association was robust to multiple sensitivity analyses designed to address potential partial volume effects and regional specificity, including adjustment for tissue composition within the LC mask, the use of eroded and dilated LC masks, and comparison against the neighboring dorsal pontine control region. Furthermore, LC volume statistically mediated the relationship between age and WMHs, suggesting that individual differences in noradrenergic decline may account for a substantial portion of age‐related white matter vulnerability. Beyond total lesion load, the regional distribution of WMHs is aligned with cortical expression of ADRB2, particularly in individuals with more pronounced LC degeneration.

The first and most informative PLS pattern (PLS1), a composite score dominated by LC volume, SN volume, and age, consistently mapped the brain regions most prone to white matter lesions across participants. This spatial pattern showed a significant overlap with cortical ADRB2 expression. Moreover, the association between this PLS‐derived WMH pattern and total lesion burden was statistically mediated by ADRB2. Together, these findings suggest that regional noradrenergic sensitivity may shape how LC‐related vulnerability is expressed across the cortex in the aging brain, indicating that LC atrophy is linked not only to the overall burden of WMHs but also to their spatial distribution.

A converging body of evidence identifies that astrocytic ADRB2s act as a key regulatory node for metabolic support.^24^, ^26^, ^41^ Activation of these receptors triggers glycogenolysis and rapid lactate export, which is taken up by myelinated axons via MCT2 to fuel oxidative ATP production.^42^, ^43^, ^44^, ^45^ Disruption of β_2_ signaling suppresses this lactate surge^24^, ^41^ and attenuates astrocytic reactivity, including reduced glial fibrillary acidic protein (GFAP) expression—an established marker of aging and dementia.^23^, ^46^, ^47^ Importantly, β_2_‐mediated metabolic support appears to be dynamically modulated by LC activity rather than operating as a static on/off mechanism. While LC stimulation can transiently increase cortical excitability through β_2_ receptors over minutes,^48^ age‐related LC degeneration or systemic β‐blockade uncouples lactate release from neuronal demand.^24^, ^49^ This suggests a state‐dependent failure of noradrenergic control, in which reduced tonic LC input leads to basal hypometabolism and relative hypoexcitability. Yet, residual phasic LC responses—such as those elicited by stress or arousal—may still provoke exaggerated excitatory states that are no longer adequately supported metabolically.

Such a mismatch between neuronal activity and astrocytic energy supply offers a potential framework to reconcile an LC‐dependent hypometabolic signature of aging,^50^ with evidence for cortical hyperexcitability and altered excitation–inhibition balance in later life.^1^, ^51^, ^52^, ^53^ Consistent with this view, PET studies show that regions destined to develop WMHs exhibit low resting glycolysis in healthy older adults, followed by paradoxical hypermetabolic responses once small‐vessel disease emerges, suggestive of chronic energy debt and compensatory astrocytic activation.^9^ Under this model, β_2_‐adrenergic regulation of astrocytic glycogenolysis functions less as a fixed supply line and more as a gain control whose calibration progressively deteriorates with aging—blunted at rest, exaggerated during demand, and ultimately insufficient to sustain white matter integrity.

Our finding that LC atrophy predicts both the burden and spatial distribution of WMHs is consistent with evidence from age‐related neurodegenerative disease. While LC volume showed robust associations across analyses, we note that NBM volume was also significantly associated with q‐WMH burden. The NBM is a major source of cortical cholinergic innervation and is known to undergo early degeneration in Alzheimer's disease, preceding and predicting the cortical spread of pathology,^54^ and volumetric approximations have reproduced this phenomenon.^55^ This effect, however, was not observed for manually delineated WMHs, suggesting that cholinergic contributions may be more sensitive to diffuse or probabilistic lesion estimates rather than focal lesion burden.

β_2_‐adrenergic transcripts and receptor binding are markedly reduced in multiple sclerosis plaques and surrounding astrocytes,^25^, ^26^ and pharmacological β_2_ antagonism exacerbates experimental demyelination.^27^, ^56^ Recent work extends this to cognition: astrocyte‐specific β_2_ receptors mediate LC‐dependent enhancement of both recent and remote memory, an effect abolished when lactate export is blocked.^48^, ^57^, ^58^ These data position β_2_‐driven lactate shuttling as a shared metabolic currency for myelin maintenance, plasticity, and memory. We propose that LC degeneration removes the noradrenergic “dial” that scales this shuttle to fluctuating axonal demand, where regional β_2_‐receptor density is high, the loss is felt most acutely, producing focal energy failure and the demyelination signature captured as WMHs. The β_2_–WMH spatial coupling observed in our cohort, therefore, represents a metabolic vulnerability map that may serve as a biomarker for targeted β_2_‐adrenergic or lactate‐augmenting interventions.

Several limitations should be acknowledged. LC volume was estimated using VBM and a mask derived from neuromelanin‐sensitive MRI templates rather than direct neuromelanin imaging, and we did not directly measure noradrenaline levels or astrocytic metabolic activity; accordingly, the proposed mechanism is supported by indirect anatomical and spatial associations rather than direct physiological evidence. The cross‐sectional design further precludes establishing temporal precedence, such that we cannot determine whether LC atrophy precedes WMH development or reflects a parallel process during aging. In addition, ADRB2 expression was assessed using population‐level cortical maps rather than subject‐specific measures, limiting inference about individual variability in noradrenergic sensitivity. Although participants were clinically nondemented, we did not assess amyloid‐β or tau pathology, which can accumulate decades before symptoms and for which the LC is an early site of involvement, raising the possibility that subclinical Alzheimer's disease processes contributed to the observed associations. Finally, while the sample size was modest, we mitigated potential biases related to LC measurements, and the effects were statistically robust, consistent across cohorts (ANDES, ADNI), and biologically coherent, surviving correction for multiple comparisons and spatial autocorrelation.

## AUTHOR CONTRIBUTIONS

*Conceptualization*: Vicente Medel. *Methodology*: Victor Vidal, Vicente Medel, Gabriel Wainstein, Patricia Orellana, James M. Shine, and Robert D. Sanders. *Software and analysis*: Victor Vidal, Vicente Medel, Raul Gonzalez‐Gomez, and Gabriel Wainstein. *Data curation*: Carolina Delgado, Patricia Orellana, Raul Gonzalez‐Gomez, and Rodrigo C. Vergara. *Writing—original draft*: Vicente Medel and Victor Vidal. Writing—review and editing: all authors. *Supervision*: Gonzalo Farías, Carolina Delgado, Vicente Medel, James M. Shine, and Robert D. Sanders. *Funding acquisition*: Vicente Medel, Carolina Delgado, Paul H. Delano, Gonzalo Farías, James M. Shine, and Robert D. Sanders.

## CONFLICT OF INTEREST STATEMENT

The authors declare no conflicts of interest.Author disclosures are available in the Supporting Information.

## CONSENT STATEMENT

All participants provided written informed consent following the Declaration of Helsinki, and the study was approved by the local Institutional Review Board.

## Supporting information




**Supporting Information**: alz71583‐sup‐0001‐SupMat.docx

**Supporting Information**: alz71583‐sup‐0002‐ICMJE.pdf
