# Supplementary material for: White matter hyperintensities are associated with locus coeruleus atrophy and astrocytic β2‐adrenergic receptor expression
Source: Alzheimers Dement. 2026 Jun 17;22(6):e71583. doi: 10.1002/alz.71583 (PMC13275325; doi:10.1002/alz.71583)
Supplement: Supplementary file 1 — Supporting Information: alz71583‐sup‐0001‐SupMat.docx [file ALZ-22-e71583-s002.docx]

Supplementary Materials

**White matter hyperintensities are associated with locus coeruleus atrophy and astrocytic β_2_-adrenergic receptor expression**

Victor Vidal^1^*, Gonzalo Farías^2,3^ *, Carolina Delgado^2,3^, Paul H. Delano^2, 3^, Rodrigo C. Vergara^4,5^, Patricia Orellana^3^, Tomás Ossandón^6^, Nicolás A. Crossley^6^, Cecilia Gonzalez-Campo^7^, Sharon L. Naismith^1^, Raul Gonzalez-Gomez^8^, Carlos Coronel-Oliveros^8, 9^, Agustín Ibáñez^7,8,9,10,11^, Gabriel Wainstein^12^, Robert D. Sanders^1^, James M. Shine^1^, and Vicente Medel^13^

^1^ Brain and Mind Centre, The University of Sydney, Sydney, Australia

^2^ Departamento de Neurociencia, Facultad de Medicina, Universidad de Chile, Santiago, Chile

^3^ Hospital Clínico de la Universidad de Chile, Santiago, Chile

^4^ Departamento de Kinesiología, Universidad Metropolitana de Ciencias de la Educación, Santiago, Chile.

^5^ Centro Nacional de Inteligencia Artificial (CENIA), Santiago, Chile.

^6^ Departamento de Psiquiatría, Facultad de Medicina, Pontificia Universidad Católica de Chile, Santiago, Chile

^7^ Centro de Neurociencia Cognitiva, Universidad de San Andrés, Buenos Aires, Argentina

^8^ Latin American Brain Health Institute (BrainLat), Universidad Adolfo Ibanez, Santiago, Chile

^9^ Global Brain Health Institute (GBHI), University of California, US, and Trinity College Dublin, Ireland

^10^ Department of Biophysics, School of Medicine, Istanbul Medipol University, Istanbul, Türkiye

^11^ Barcelonaβeta Brain Research Center (BBRC), Pasqual Maragall Foundation, Barcelona, Spain

^12^ Independent Researcher, Sydney, Australia

^13^ Facultad de Ciencias Biológicas, Pontificia Universidad Católica de Chile, Santiago, Chile

**These authors share first authorship*

**Correspondence to:**

Vicente Medel, PhD

Facultad de Ciencias Biológicas, Pontificia Universidad Católica de Chile

Avenida Libertador Bernardo O’Higgins 340, Santiago 8331150, Chile

Email: [vimedel@uc.cl](mailto:vicente.medel@gmail.com)

**Supplementary Methods**

**Sensitivity analyses using alternative LC ROI definitions:** To evaluate the robustness of the LC–WMH association to ROI definition and potential partial-volume effects, we conducted sensitivity analyses using eroded and dilated versions of the LC mask.

The original bilateral LC mask derived from the MetaMask atlas contained 76 voxels (41 left, 35 right) at the resolution of the normalized gray matter maps (1 mm isotropic). Because the LC has a very small anatomical cross-section, applying a standard voxel-wise erosion at native resolution would substantially reduce the ROI. To perform a conservative erosion while preserving anatomical continuity, the LC mask was first upsampled to 0.5 mm isotropic resolution, after which a 1-voxel spherical morphological erosion was applied. The eroded mask was then resampled back to the native 1 mm resolution used for VBM analyses. This procedure yielded a conservative LC core mask containing 5 voxels, representing the central portion of the ROI where partial-volume effects from surrounding tissue are minimized.

In addition, a dilated LC mask was generated by expanding the original LC ROI by one voxel in all directions, producing a larger mask containing 255 voxels that intentionally included adjacent tissue. This dilation analysis tested whether the observed LC–WMH association could be explained by signal leakage from neighboring white matter or surrounding brainstem structures.

Gray matter signal estimates were extracted from both the eroded and dilated masks using the same VBM-derived modulated gray matter maps used in the primary analyses. Regression models identical to those used in the main analysis were then repeated using these alternative ROI definitions.

**Supplementary Figures**

**Figure S1. Tissue composition and age associations within the LC.**
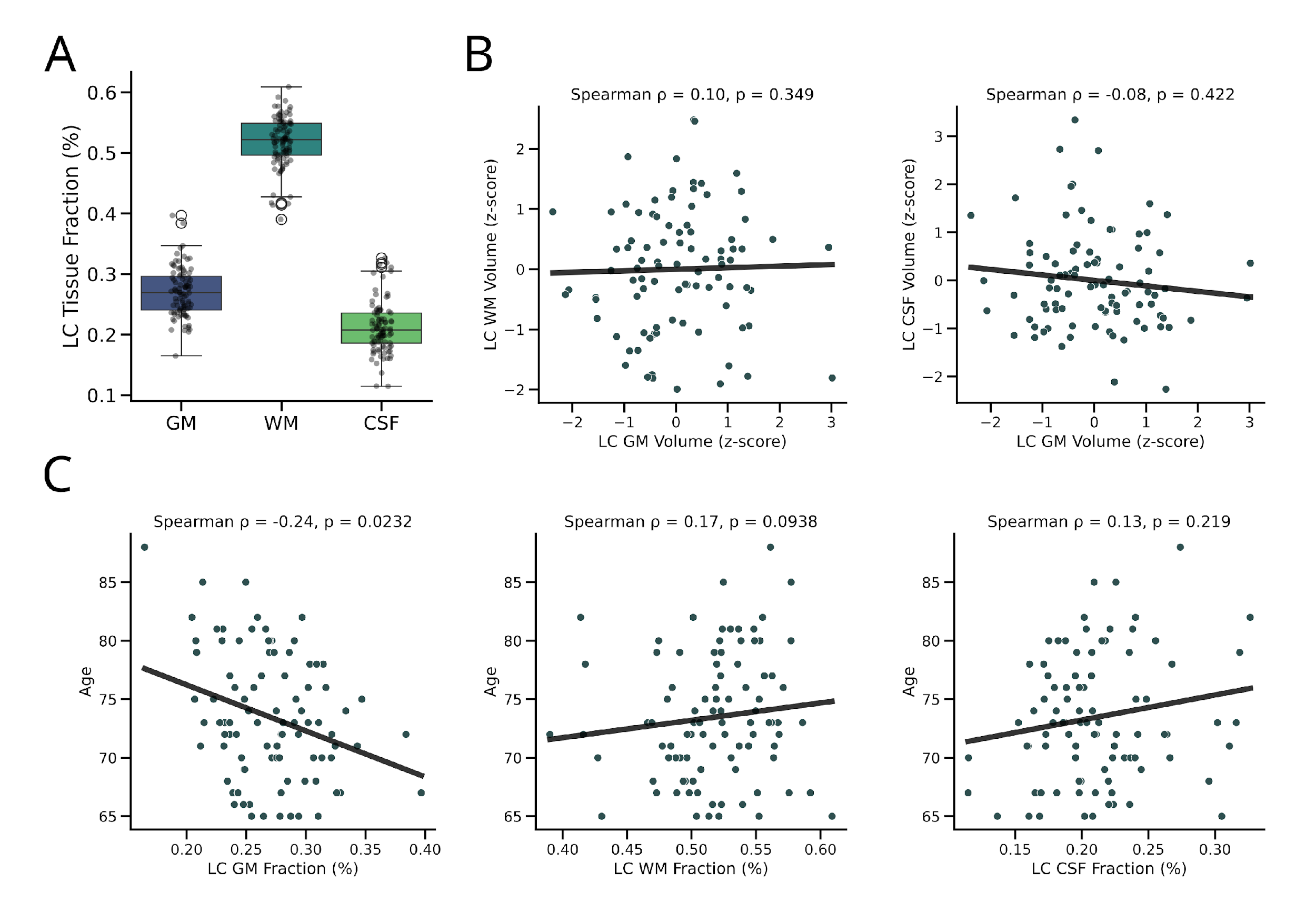
(A) Distribution of probabilistic tissue fractions within the LC ROI across subjects. (B) Relationship between LC gray matter volume and LC white matter (left) and CSF (right) signal estimates. No significant correlations were observed (Spearman ρ = 0.10, p = 0.349; ρ = −0.08, p = 0.422), indicating that LC GM estimates are not strongly coupled with local WM or CSF fractions within the ROI. (C) Associations between tissue fractions within the LC mask and age. LC gray matter fraction decreased significantly with age (left, Spearman ρ = −0.24, p = 0.023), whereas LC white matter (middle) and CSF (right) fractions were not significantly associated with age (ρ = 0.17, p = 0.094; ρ = 0.13, p = 0.219).


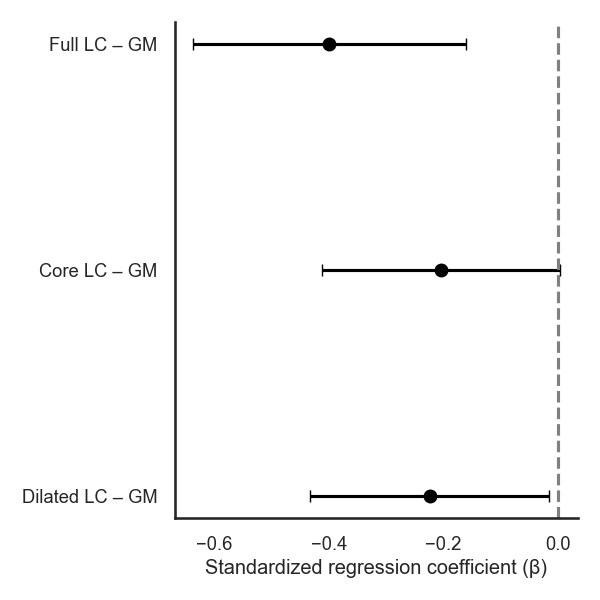


**Figure S2**. **Sensitivity of the LC–WMH association to ROI definition.** Forest plot showing standardized regression coefficients (β) and 95% confidence intervals for the association between locus coeruleus (LC) gray matter signal and WMH burden across alternative LC mask definitions, including age, intracranial volume, education, cardiovascular risk score, and sex as covariates. The full LC mask, an eroded LC core mask, and a dilated LC mask were analyzed.

**Figure S3. Specificity of the LC-WMH association relative to a dorsal pontine control region.**
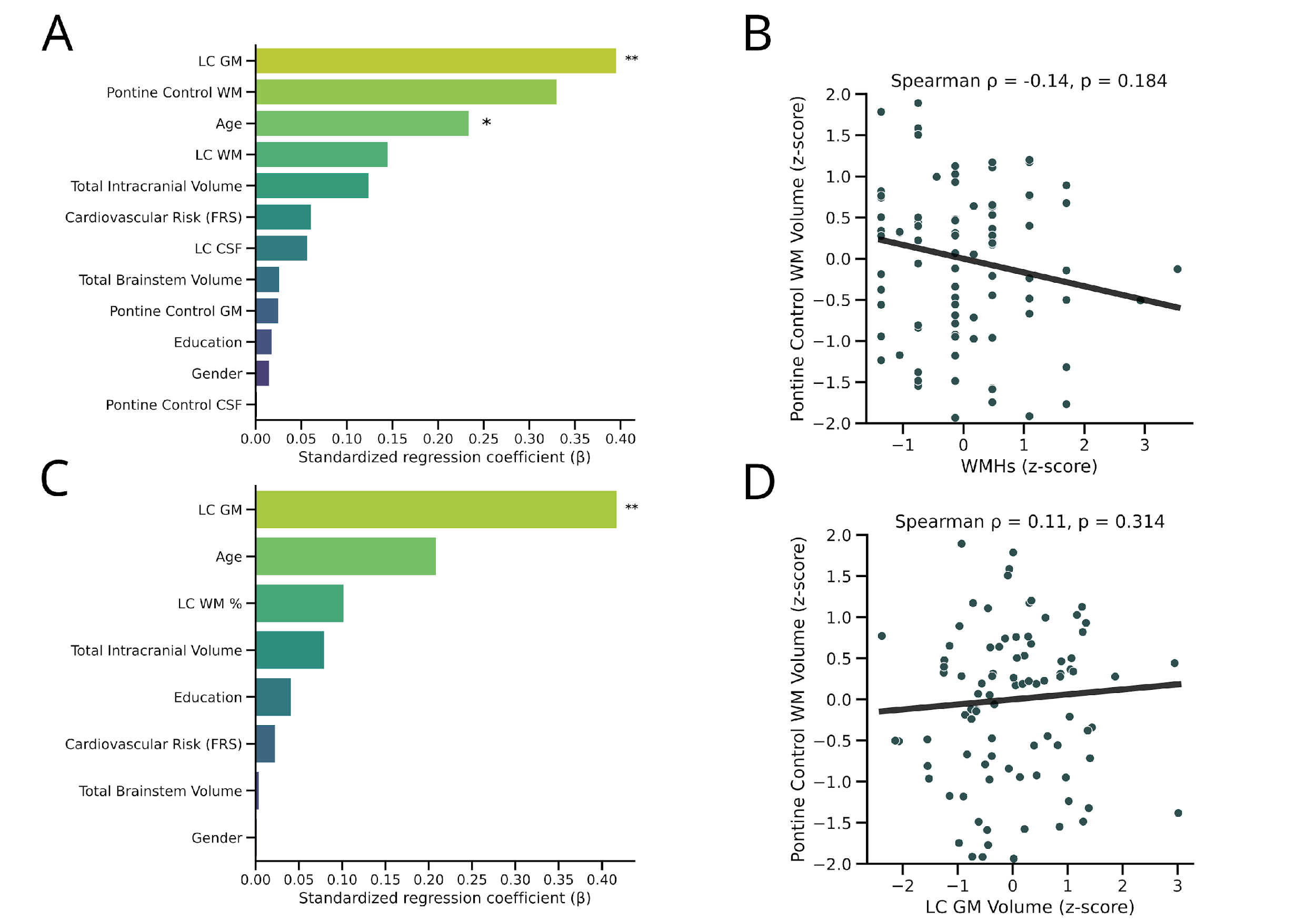
(A) Standardized regression coefficients (β) from a multivariable model including the LC GM signal together with a dorsal pontine control ROI (GM, WM, CSF) and demographic covariates. LC GM remained significantly associated with WMH burden, whereas the pontine control region measures were not associated with WMH load, although a medium non-significant effect size was found for Pontine Control WM. (B) Scatter plot showing the relationship between WMH burden and white matter signal extracted from the pontine control ROI. No significant association was observed (Spearman ρ = −0.14, p = 0.184). (C) Regression model testing whether the LC-WMH association persists after accounting for local white matter fraction within the LC mask. LC GM remained significantly associated with WMH burden, whereas LC WM fraction was not significant. (D) Relationship between the LC GM signal and the white matter signal from the pontine control ROI. No significant correlation was observed (Spearman ρ = 0.11, p = 0.314), indicating that LC GM estimates are not driven by neighboring pontine white matter signal.

**Figure S4. Replication of the LC–WMH association in the ADNI cohort (n=126) age- and gender-matched healthy controls.**
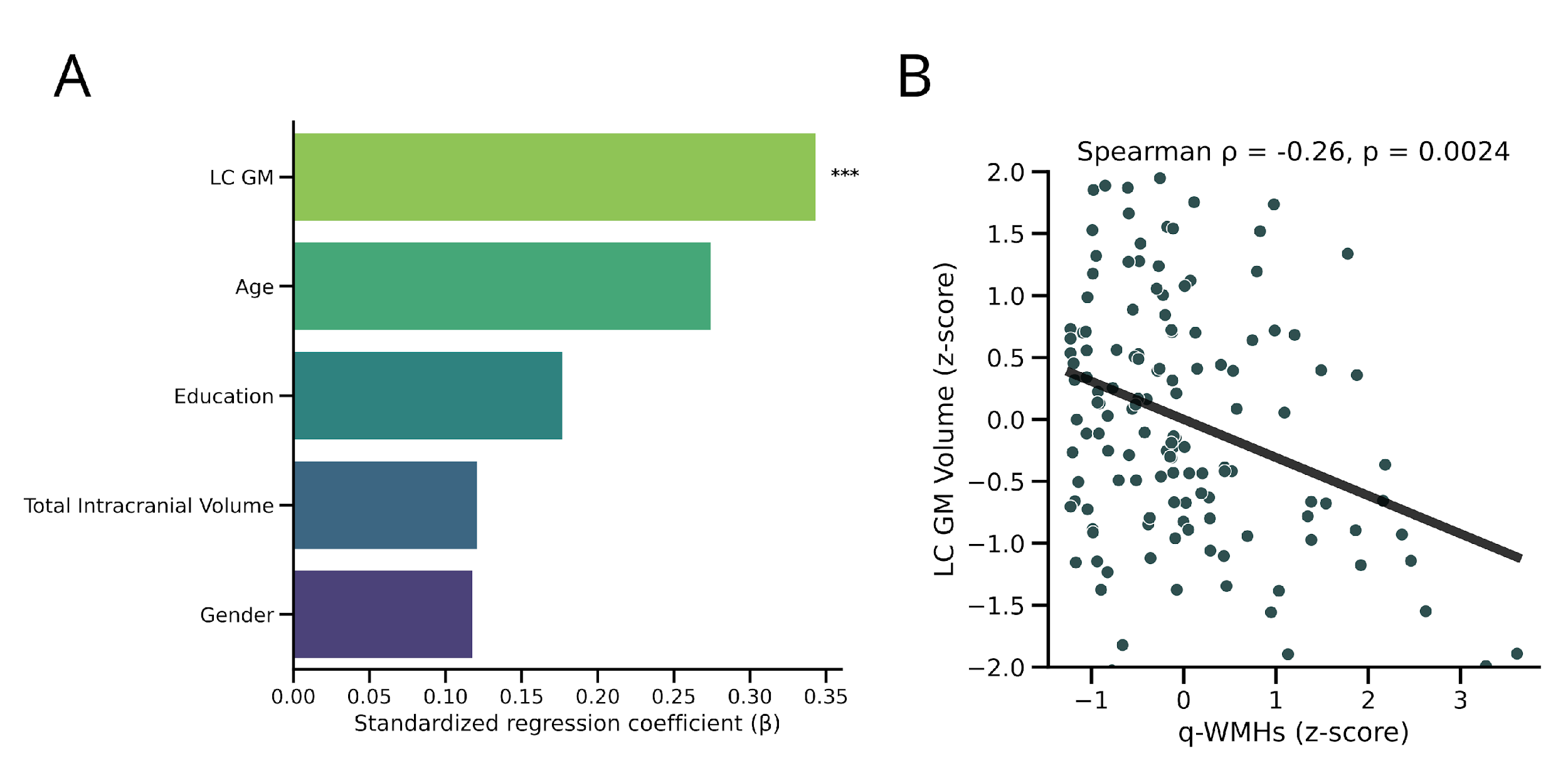
(A) Standardized regression coefficients (β) from a multivariable model predicting q-WMH burden, including LC gray matter (GM) volume, age, sex, education, and total intracranial volume. LC GM volume showed the largest effect size in the model. (B) Scatter plot showing the association between LC GM volume and WMH burden (z-scores). A significant negative correlation was observed (Spearman ρ = −0.26, p = 0.0024).


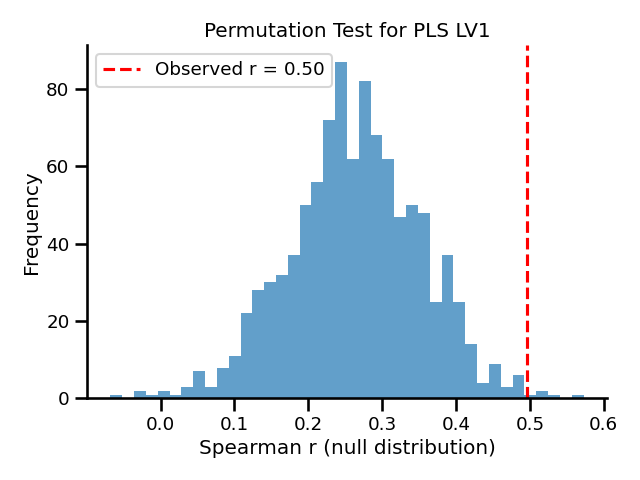


**Figure S5.** The observed correlation between PLS1 scores from q-WMHs and anatomical/demographical single-subject information was r = 0.5, which was significantly greater than expected by chance (empirical *p* < 0.001, based on 1,000 permutations).

| ***ROI (voxels)*** | ***Tissue (%)*** | ***β (standardized)*** | ***95% CI*** | ***p*** |
| --- | --- | --- | --- | --- |
| Locus Coeruleus      (*76*) | GM (*27*) | −0.402 | −0.637 – −0.167 | **0.001 **** |
|  | WM *(51.8)* | −0.166 | −0.370 – 0.038 | 0.110 |
|  | CSF *(21.2)* | −0.056 | −0.268 – 0.157 | 0.604 |
| Basal Forebrain (*368*) | GM *(52)* | −0.211 | −0.523 – 0.100 | 0.180 |
|  | WM *(25)* | 0.025 | −0.292 – 0.342 | 0.875 |
|  | CSF *(23)* | 0.163 | −0.142 – 0.467 | 0.292 |
| Substantia Nigra (*152*) | GM *(7)* | −0.218 | −0.468 – 0.045 | 0.105 |
|  | WM *(85)* | −0.197 | −0.814 – 0.420 | 0.528 |
|  | CSF *(8)* | 0.150 | −0.137 – 0.436 | 0.302 |

**Table S1**. Voxel counts, tissue composition, variance metrics, and standardized regression effect sizes for each ROI predicting WMHs. Identical regression models were estimated separately for each ROI to allow direct comparison of standardized effect sizes across regions while avoiding multicollinearity between regional tissue fractions.
